# Complete chloroplast genome of *Paris polyphylla* Smith var. *stenophylla* Franch.:genomic features and phylogenetic analysis

**DOI:** 10.1080/23802359.2026.2685910

**Published:** 2026-06-13

**Authors:** Qing-Dong Ma, Hong-Jing Zhang, Xiao-Xu Zhou, Ya Huang, Hong-Yuan Chen, Si-Rong Yi

**Affiliations:** Chongqing Key Laboratory of Development and Utilization of DaoDi Medicinal Materials in Three Gorges Reservoir Area, Chongqing Three Gorges Medical College, Chongqing, P.R. China

**Keywords:** *Paris polyphylla* var. *stenophylla*, chloroplast genome, phylogenetic tree

## Abstract

*Paris polyphylla* Smith var. *stenophylla* Franch. (1888) is recognized in the medicinal standards of Gansu and Zhejiang provinces. We report its complete chloroplast genome, which is 164,767 bp long and has a typical quadripartite structure including an LSC region of 84,296 bp, an SSC region of 12,955 bp, and two IRs of 33,758 bp each. The genome contained 134 genes, including 113 unique genes.. Phylogenetic analysisplaced itas a sister to *P. caojianensis* and.close to *P. polyphylla* var. *yunnanensis.* These findings provide genomic resources for future studies of this medicinal herb.

## Introduction

Paridis rhizoma, known as ‘Chonglou’, has long been used in traditional Chinese medicine for treating boils and abscesses, sore throat, snake and insect bites, traumatic injuries from falls, and convulsions with wind-heat syndrome (Zhang et al. 2025). There are about 33 species and 15 varieties of the *Paris* genus (Hua et al. 2025); 22 of those (12 endemic) present in China. The medicinal plants in Paridis rhizoma consist primarily of *Paris polyphylla* Smith var. *yunnanensis* (Franch.) Hand.-Mazz. (1936) and *P. polyphylla* Smith var. *chinensis* (Franch.) Hara (1969), both documented in the *Chinese Pharmacopeia* since 1977 (Gu et al. 2024). Furthermore, in folk medicine practices, Paridis rhizoma has historically been employed as a broad term to encompass closely related species, consistent with the documented botanical diversity in herbal textual research (Li et al. 2023).

**Figure 1.**
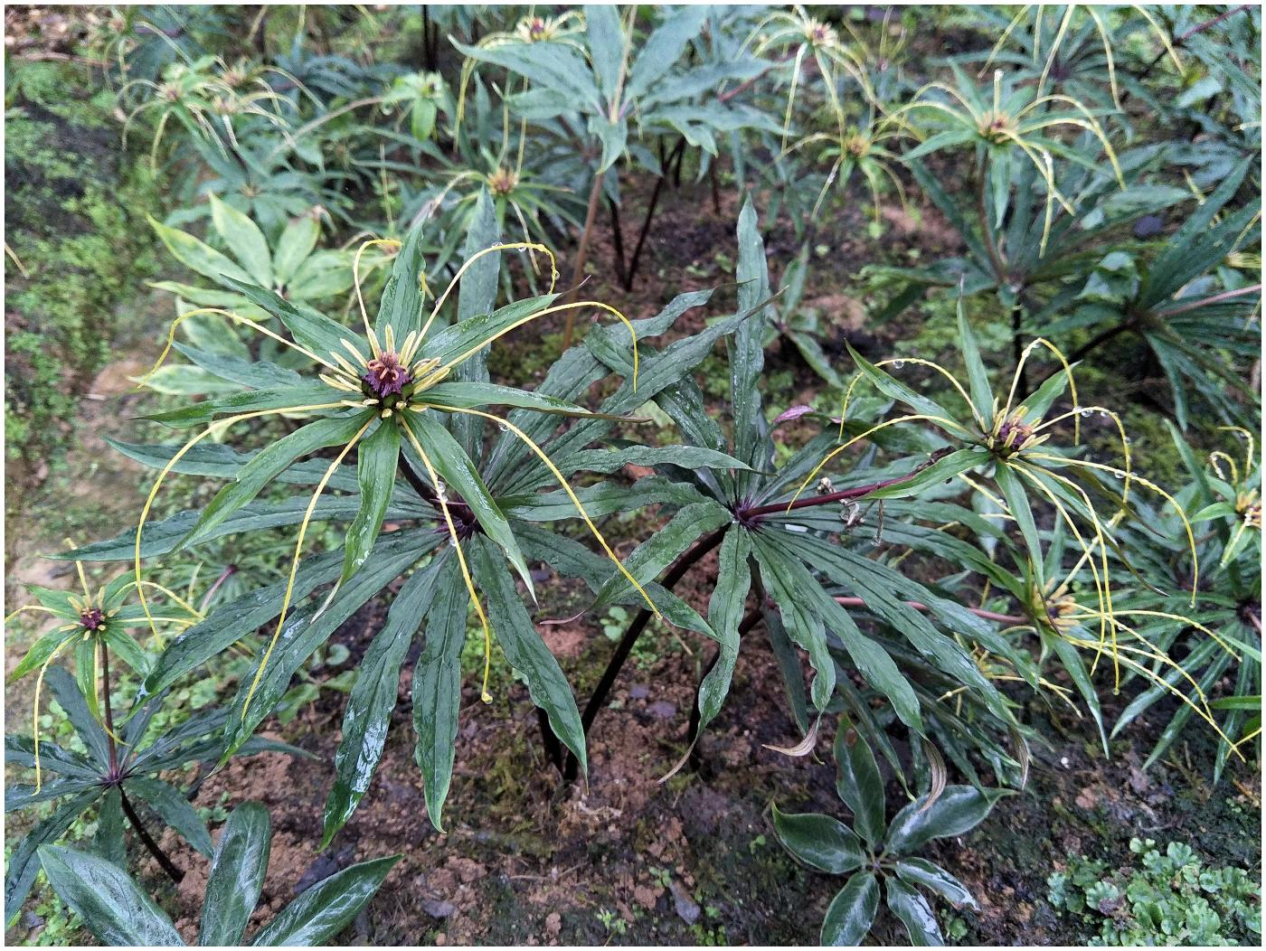
Photograph of *Paris polyphylla* var. *stenophylla* at Wanyuan (N32°12′47′′, E108°14′60′′), Sichuan, China. Core features: Plants 35–115 cm tall. Rhizome 1–2.5 cm thick, leaves (6-)8–14(−22); leaf blade 6–15(−30) × 1.5–2.5 cm, base rounded to cuneate, peduncle 5–24(−65) cm, petiole (0.5-)1–6 cm, sepals (3 or) 4–6 (or 7), narrowly ovate-lanceolate to lanceolate, (3-)4.5–7(−11) × 1–4 cm, the petals narrowly linear, (3-) 4.5–7 (−11) cm × 1–2 mm, anthers 5–6 mm; free portion of connective *circa* 0.5 mm, ovary ribbed, sometimes tuberculate, style purple to white; stigma lobes (4 or) 5, capsule globose, sometimes tuberculate.

However, due to China’s vast territory, rich biodiversity, and long history of local herbal traditions, pharmacopeia standards alone do not fully capture actual folk usage, where a single herbal name may encompass multiple species. For Paridis rhizoma, one such locally used species is *P. polyphylla* var. *stenophylla* Franch. (1888; Figure 1), which exhibits a wide distribution across regions from Southwest to East China, including Xizang, Sichuan, Hubei, and Zhejiang. This species holds significant medicinal value, being officially recognized as Paridis rhizoma (‘Dengtaiqi’ or ‘Zhechonglou’) in the Chinese crude drug standards of Gansu and Zhejiang Provinces (GanSu Medical Products Administration 2021; ZheJiang Medical Products Administration 2017). Such official recognition in even two provinces is noteworthy, given that among the more than 2200 locally used medicinal herbs officially recorded in provincial standards (regional standards for traditional Chinese medicinal materials), over 80% are documented in only one provincial standard, whereas only about 10% are included in two (Wu et al. 2024). This reflects region-specific medicinal practices and highlights the need for further investigation of this species. Pharmacological studies have validated its therapeutic potential, revealing multiple kinds of saponin compounds with demonstrated antitumor activity (Xiao et al. 2012; Hu et al. 2022). Phylogenetic analyses present a complex phenomenon. While infrared spectroscopy-based hierarchical cluster analysis showed that it was closely related to *P. polyphylla* var. *yunnanensis* (Zhao et al. 2019), ITS2-based neighbor-joining tree analysis supported its classification as a *P. polyphylla* var. *chinensis* variant (Ye et al. 2017). Despite its ecological and pharmacological significance, genomic data for this species remain limited. To address this gap, we sequenced its complete chloroplast genome to clarify evolutionary relationships within the genus and provide foundational data for future research.

## Materials and methods

Fresh leaves of *P. polyphylla* var. *stenophylla* were collected from a traditional Chinese medicine herb farm in Wanyuan, Sichuan, China (N32°12′47′′, E108°14′60′′). All specimens were deposited in the herbarium of Chongqing Three Gorges Medical College (YSR3196; Chongqing, China; Si-Rong Yi; yisirong123@aliyun.com). Total genomic DNA was extracted using a modified CTAB protocol. Whole-genome sequencing was performed on the BGI DNBSEQ platform, generating 9.9 GB of high-quality clean data. The complete chloroplast genome was assembled with GetOrganelle v1.7.7.0 (Jin et al. 2020) using the chloroplast genome of *P. caojianensis* (MZ147601) as reference (Jiang et al. 2022). Initial genome annotation was performed with CPGAVAS2, followed by manual curation using CPStools (Huang et al. 2024). The circular genome map and gene structure visualization, including *cis*- and *trans*-splicing genes, were constructed using CPStools, which was also used to identify simple sequence repeats (SSRs) with the following minimum repeat thresholds: 10 for mononucleotides, 6 for dinucleotides, 5 for trinucleotides, 5 for tetranucleotides, 5 for pentanucleotides, and 5 for hexanucleotides (k 10,6,5,5,5).

To determine the phylogenetic location of *P. polyphylla* var. *stenophylla*, the complete chloroplast genome sequences of 24 *Paris* species were retrieved from the NCBI GenBank database. *Trillium tschonoskii* (KR780076) and *T. camschatcense* (MN125568) were selected as outgroups. Multiple sequence alignment was performed using MAFFT, and ambiguously aligned regions were removed using trimAl with a gap threshold of 0.8 (Katoh and Standley 2013). The optimal nucleotide substitution model (GTR+G + I) was determined using ModelTest-NG. A maximum likelihood (ML) phylogenetic analysis was conducted using RAxML (Stamatakis 2014) with 1,000 bootstrap replicates to assess branch support.

## Results

**Figure 2.**
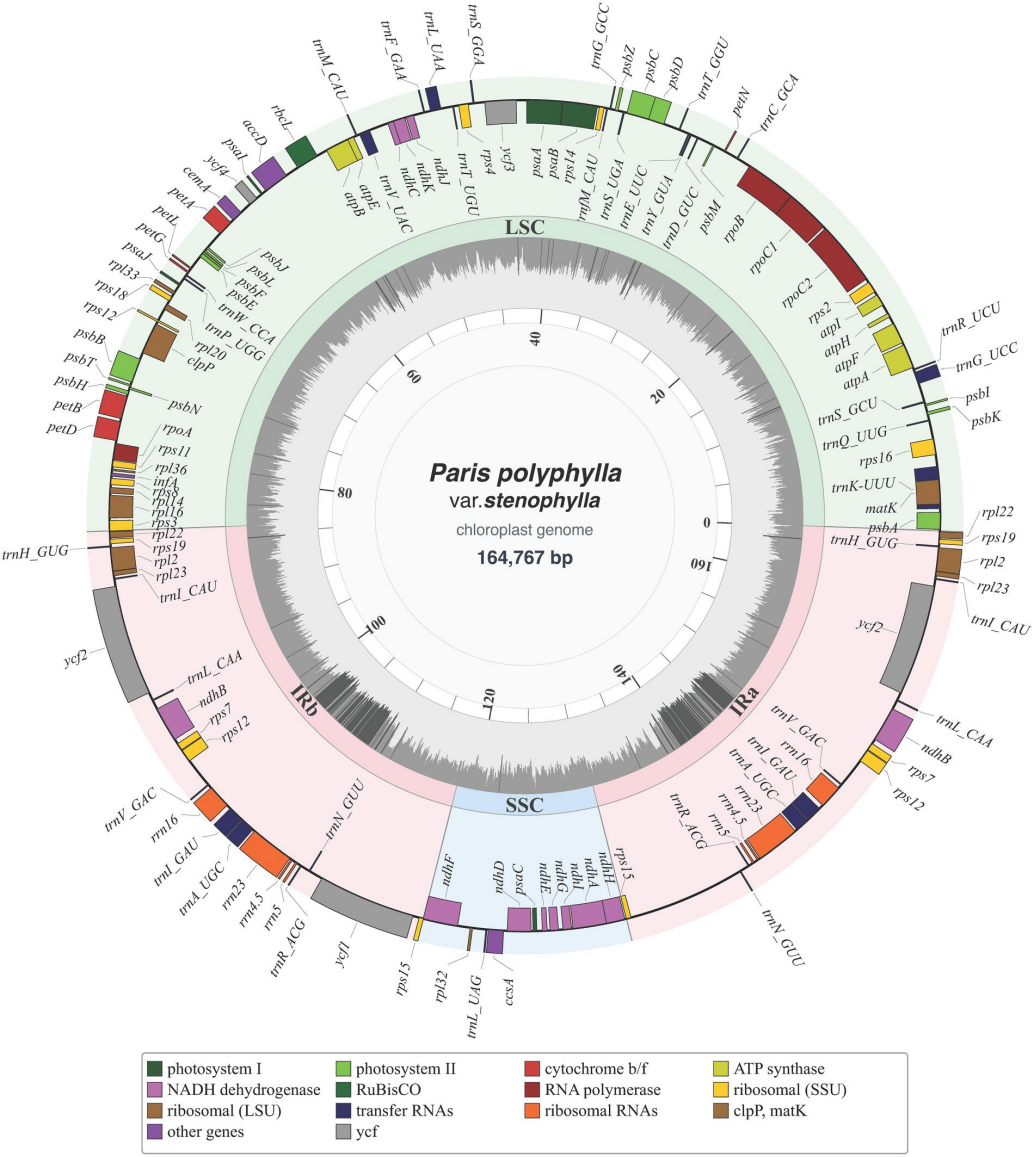
Chloroplast genome map of *Paris polyphylla* var. *stenophylla*. The chloroplast genome is characterized by a quadripartite structure, including a large single-copy (LSC) region, a small single-copy (SSC) region, and two inverted repeats flanking them. The color scheme for genes corresponds to their functional classification, as specified below the figure.

The chloroplast genome of *P. polyphylla* var*. stenophylla* was successfully assembled with an average sequencing depth of 1,873×; most nucleotide positions were covered by > 1,000 reads (Figure S1), confirming the high accuracy of the assembly. The *cis-* and *trans-*splicing gene structure map further validated the annotation accuracy (Figure S2). The complete chloroplast genome is 164,767 bp in length, exhibiting the typical quadripartite structure characteristic of angiosperm chloroplast genomes (Figure 2). It comprised a large single-copy (LSC) region of 84,296 bp, a small single-copy (SSC) region of 12,955 bp, and two inverted repeats (IRs) of 33,758 bp each, separating them. A total of 134 genes were annotated, including 113 unique genes, of which 88 were protein-coding genes (79 unique), 38 tRNA genes (30 unique), and 8 rRNA genes (4 unique; Table S1). The overall guanine–cytosine (GC) content was 36.9%, with notable regional variation: IR regions exhibited the highest GC content (39.4%), followed by the LSC region (35.6%), with the SSC region showing the lowest (32.1%). Functional classification assigned the annotated genes to three major categories: photosynthesis-related genes, self-replication genes, and genes with other functions (Table S2). Eleven protein-coding genes contained introns: nine (*ndhA*, *ndhB*, *petB*, *petD*, *atpF*, *rpoC1*, *rps16*, *rpl2*, and *rpl16*) harbored a single intron each, whereas two (*clpP* and *ycf3*) contained two. Notably, *rps12* was identified as a *trans*-spliced gene, with its 5′ exon located in the LSC region and 3′ exons in IR regions. In addition, eight protein-coding genes (*ndhB*, *rps7*, *rps15*, *rps19*, *rpl2*, *rpl22*, *rpl23*, and *ycf2*), four rRNA genes (*rrn4.5*, *rrn5*, *rrn16*, and *rrn23*), and eight tRNA genes (*trnA-UGC*, *trnH-GUG*, *trnI-CAU*, *trnI-GAU*, *trnL-CAA*, *trnN-GUU*, *trnR-ACG*, and *trnV-GAC*) were duplicated in IR regions.

A total of 82 SSRs were identified (Table S3), predominantly composed of mononucleotide to tetranucleotide repeats, with the former being the most abundant. A/T-rich motifs accounted for 76.83% of all SSRs.

**Figure 3.**
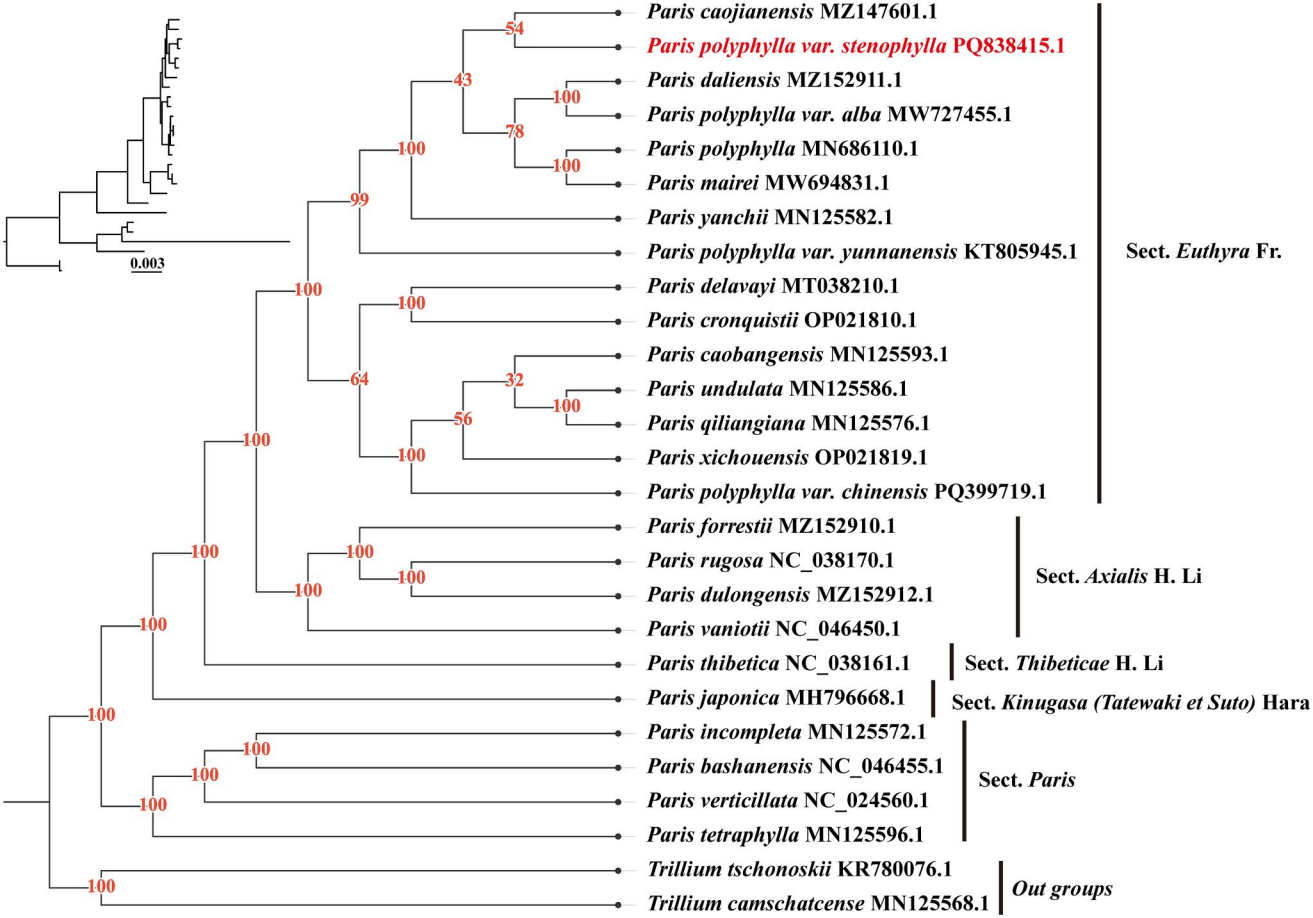
Maximum likelihood phylogenetic tree of *Paris polyphylla* var. *stenophylla* and related species. The robustness of maximum likelihood (ML) tree branches was evaluated through 1000 bootstrap replicates. The following sequences were used: *P. caojianensis* MZ147601, *P. daliensis* H. Li & V. G. Soukup (1992) MZ152911 (Jiang et al. 2022), and *P. polyphylla* var. *alba* H. Li & R. J. Mitch. (1986) MW727455 (Jiang et al. 2021a), *P. polyphylla* Sm. (1813) MN686110 (Ji et al. 2020), *P. mairei* H. Lév.(1912) MW694831 (Jiang et al. 2021b), *P. yanchii* H. Li, L.-G. Lei & Y.-M. MN125582 (Ji et al. 2019), *P. polyphylla* var. *yunnanensis* KT805945 (Song et al. 2017b), *P. delavayi* Franch.(1898) MT038210 (Ling et al., 2020), *P. cronquistii* (Takht.) H. Li (1984) OP021810 (Ji Y., 2023a), *P. caobangensis* Y.H.Ji, H.Li & Z.K. MN125593 (Ji et al. 2019), *P. undulata* H. Li & V. G. Soukup (1992) MN125586 (Ji et al. 2019), *P. qiliangiana* H. Li, J. MN125576 (Ji et al. 2019), *P. xichouensis* H. Li (1986) OP021819 (Ji Y., 2023b), *P. polyphylla* var. *chinensis* PQ399719 (Wang Y., 2024), *P. forrestii* (Takht.) H. L (1984) MZ152910 (Jiang et al. 2022), *P. rugosa* H. Li & Kurita (1992) NC_038170 (Song et al. 2017b), *P. dulongensis* H. MZ152912 (Jiang et al. 2022), *P. vaniotii* H. Lév. (1906) NC_046450 (Ji et al. 2019), *P. thibetica* Franch.(1888) NC_038161 (Song et al. 2017a), *P. japonica* Franch. (1888) MH796668 (Yang et al. 2019), *P. incompleta* M.Bieb. (1808) MN125572 (Ji et al. 2019), *P. bashanensis* F. T. NC_046455 (Ji et al. 2019), *P. verticillata* M. Bieb. (1819) NC_024560 (Do et al. 2014), *P. tetraphylla* A.Gray. (1859) MN125596 (Ji et al. 2019), *Trillium tschonoskii* Maxim. (1883) KR780076 (Kim et al. 2016) and *T. camschatcense* Ker Gawl. (1805) MN125568 (Ji et al. 2019).

The ML phylogenetic tree based on complete chloroplast genome sequences showed strong bootstrap support (Figure 3). *P. polyphylla* var*. stenophylla* showed a well-supported sister relationship with *P. caojianensis* B. Z. Duan & Y. Y. Liu (2017). Within the *Euthyra* section, *P. polyphylla* var*. stenophylla* clustered with *P. polyphylla* var*. yunnanensis* and *P. polyphylla* var*. chinensis*, with *P. polyphylla* var*. stenophylla* and *P. polyphylla* var. *yunnanensis* on the same subclade, while *P. polyphylla* var*. chinensis* occupied a distinct subclade.

## Discussion and conclusion

Chloroplast genomes are critical molecular markers for reconstructing evolutionary relationships and discriminating between closely related species (Jiang et al. 2022). The quadripartite organization, gene content (134 genes; 113 unique), and IR > LSC > SSC GC-content pattern observed in *P. polyphylla* var. *stenophylla* are all consistent with those commonly reported for angiosperm chloroplast genomes, confirming the reliability of our assembly and annotation. The A/T-rich composition of the identified SSRs (76.83%) is also in line with the AT-biased composition generally observed in plastomes of flowering plants; these SSR markers provide valuable resources for future population genetics and molecular breeding studies in *Paris* species.

The sister relationship between *P. polyphylla* var. *stenophylla* and *P. caojianensis* indicates a close evolutionary affinity. Within *Euthyra*, the presence of *P. polyphylla* var. *stenophylla* and *P. polyphylla* var. *yunnanensis* on the same subclade suggests a closer phylogenetic relationship between *P. polyphylla* var. *stenophylla* and *P. polyphylla* var. *yunnanensis* than between either and *P. polyphylla* var. *chinensis*. This topology is consistent with previous plastome-based classifications of the genus (Ji et al. 2019) and the infrared-spectroscopy-based grouping reported by Zhao et al. (2019) but differs from the ITS2-based scheme of Ye et al. (2017), which considered *P. polyphylla* var. *stenophylla* as a variant of *P. polyphylla* var. *chinensis*. The conflicting signals between nuclear ITS2 and the plastome likely reflect the different evolutionary histories of these two genomes. Future work combining nuclear and plastid markers is needed to fully resolve infraspecific relationships in the *P. polyphylla* complex.

In conclusion, this study reports the complete chloroplast genome of *P. polyphylla* var. *stenophylla*, which shows the typical quadripartite structure of angiosperm plastomes. ML phylogenetic analysis based on whole chloroplast genomes identified *P. polyphylla* var. *stenophylla* as sister to *P. caojianensis* and demonstrated its closer affinity to *P. polyphylla* var. *yunnanensis* than to *P. polyphylla* var. *chinensis*. These findings provide molecular evidence supporting the taxonomic consideration of *P. polyphylla* var. *stenophylla* as a distinct variety and offer valuable genomic resources for species identification, classification, and further research on infraspecific relationships within the genus *Paris*.

## Supplementary Material

supplementary.docx

## Data Availability

The genome sequence data that support the findings of this research are openly available in GenBank of NCBI at https://www.ncbi.nlm.nih.gov/ under the accession no. PQ838415. The associated BioProject, BioSample, and SRA numbers are PRJNA1465530, SAMN59749138, and SRR38613845.
