# Supplementary material for: Complete chloroplast genome of Paris polyphylla Smith var. stenophylla Franch.:genomic features and phylogenetic analysis
Source: Mitochondrial DNA B Resour. 2026 Jun 13;11(7):864–8. doi: 10.1080/23802359.2026.2685910 (PMC13267019; doi:10.1080/23802359.2026.2685910)
Supplement: supplementary.docx [file TMDN_A_2685910_SM4797.docx]

**Supplementary**

**Figures and Tables**


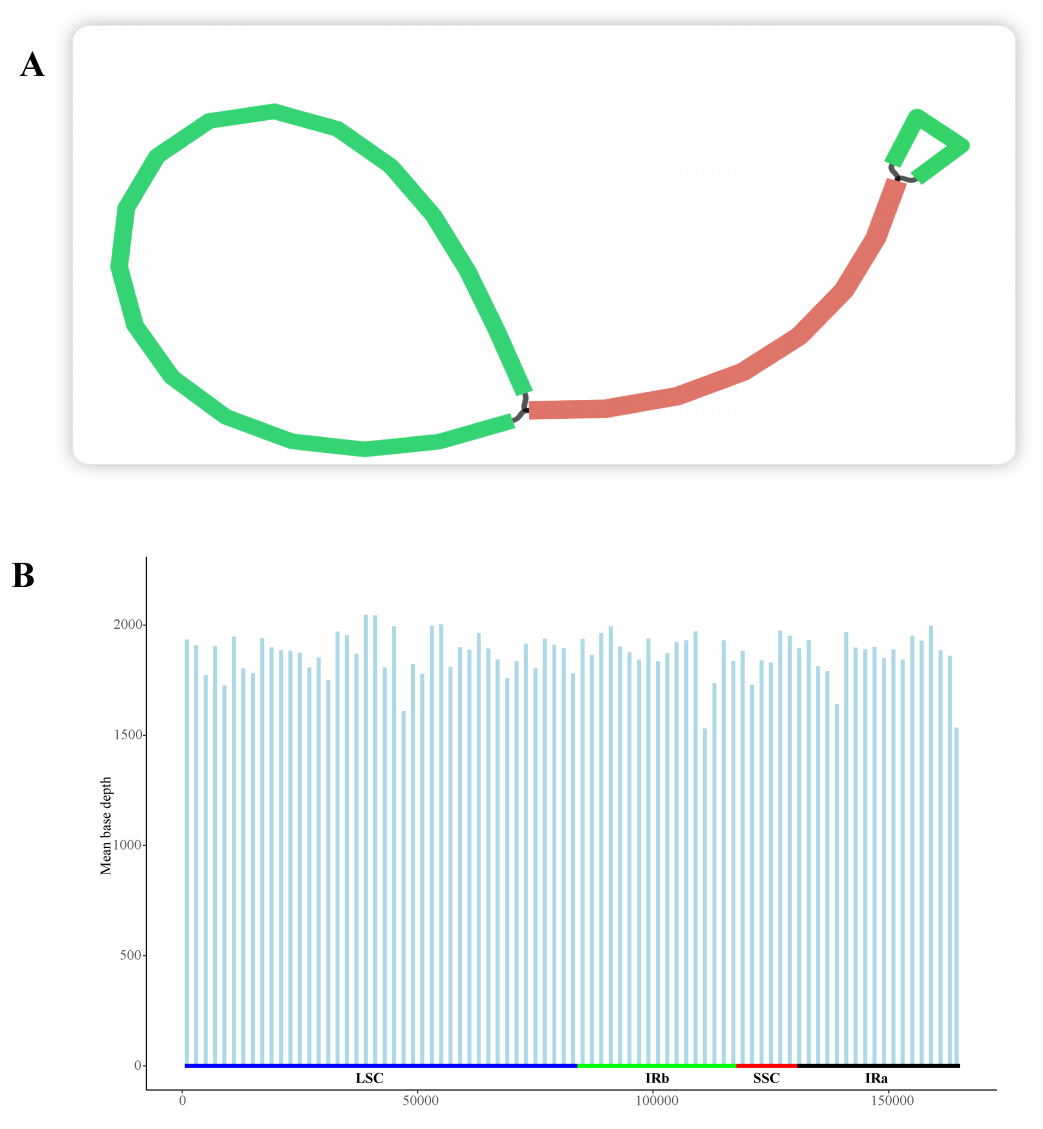


**Figure S1. Coverage depth maps of the complete chloroplast genome in *Paris polyphylla* var. *stenophylla.***


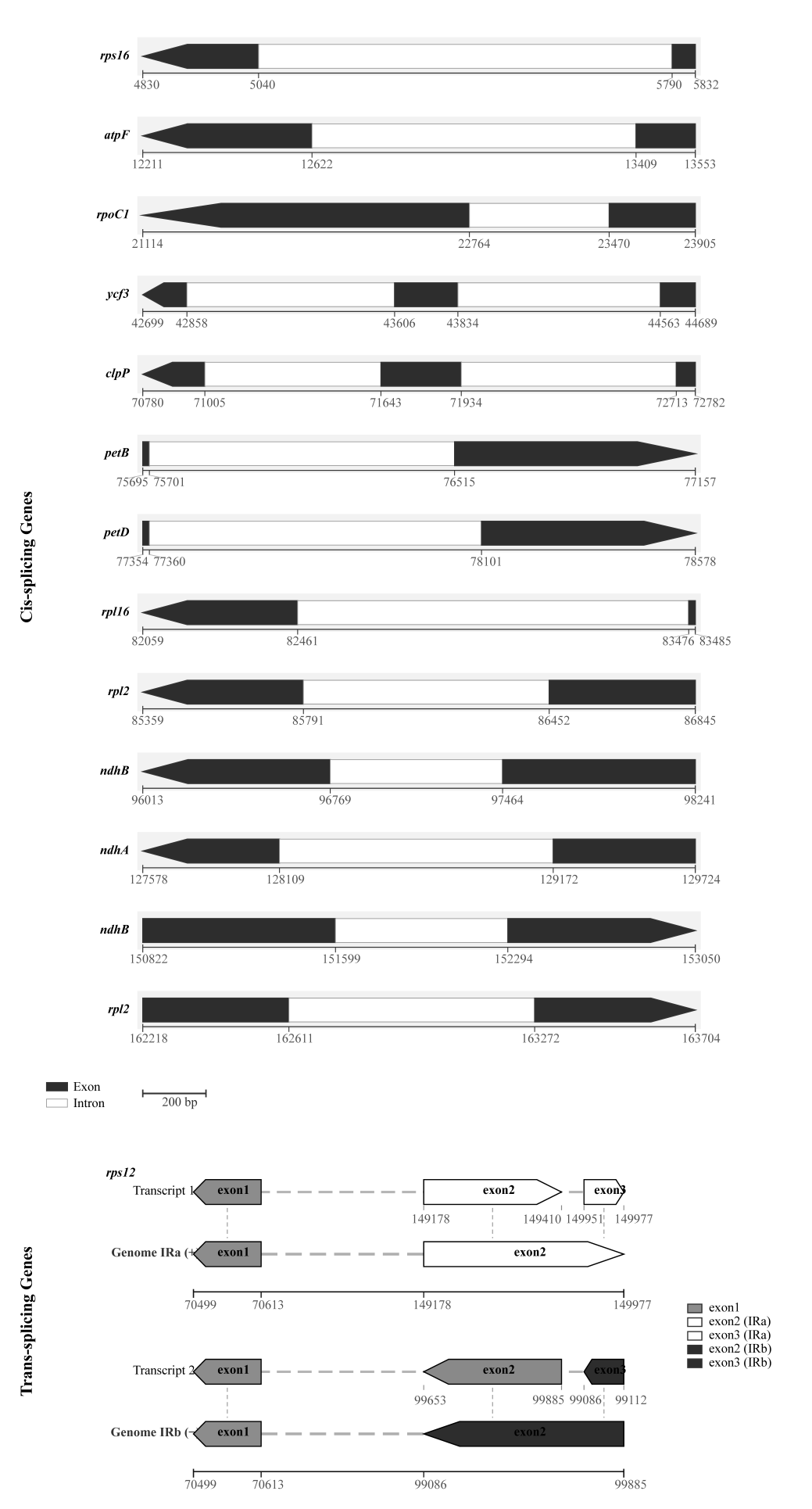


**Figure S2. Gene structure of *cis*-splicing genes and *trans*-splicing genes in the chloroplast genome of *Paris polyphylla* var. *stenophylla*. Exons are shown in black; introns in white. Arrows indicate gene sense orientation. The map was generated using CPStools.**

**Table S1 Characteristics of the complete chloroplast genomes of *Paris polyphylla* var. *stenophylla***

| **Characteristics** | | **Values** |
| --- | --- | --- |
| Length （bp） | Genome | 164,767 |
|  | LSC | 84,296 |
|  | SSC | 12,955 |
|  | IRs | 33,758 |
| Genes (unique) | Genome | 134(113) |
|  | Protein-coding | 88(79) |
|  | tRNA | 38(30) |
|  | rRNA | 8(4) |
| GC% | Genome | 36.9 |
|  | LSC | 35.6 |
|  | SSC | 32.1 |
|  | IRs | 39.4 |

Note: LSC, large single-copy; SSC, small single-copy; IRs, inverted repeats; rRNA, ribosomal ribonucleic acid; tRNA, transfer ribonucleic acid.

**Table S2. Gene functional statistics table of *Paris polyphylla* var. *stenophylla* chloroplast genome**

| **Category of genes** | **Group of genes** | **Name of genes** |
| --- | --- | --- |
| Genes for photosynthesis | Subunits of photosystem I | *psaA, psaB, psaC, psaI, psaJ* |
| Genes for photosynthesis | Subunits of photosystem II | *psbA, psbB, psbC, psbD, psbE, psbF, psbH, psbI, psbJ, psbK, psbL, psbM, psbN, psbT, psbZ* |
| Genes for photosynthesis | Subunits of cytochrome b/f complex | *petA, petB***, petD***, petG, petL, petN* |
| Genes for photosynthesis | Subunits of ATP synthase | *atpA, atpB, atpE, atpF***, atpH, atpI* |
| Genes for photosynthesis | Subunits of NADH-dehydrogenase | *ndhA***, ndhB**(×2)*, ndhC, ndhD, ndhE, ndhF, ndhG, ndhH, ndhI, ndhJ, ndhK* |
| Genes for photosynthesis | Large subunit of rubisco | *rbcL* |
| Self replication | RNA polymerase | *rpoA, rpoB, rpoC1***, rpoC2* |
| Self replication | Proteins of small subunit of ribosome | *rps2, rps3, rps4, rps7*(×2)*, rps8, rps11, rps12*(×2）*, rps14, rps15*(×2)*, rps16***, rps18, rps19*(×2) |
| Self replication | Proteins of large subunit of ribosome | *rpl2**(×2)*, rpl14, rpl16***, rpl20, rpl22*(×2)*, rpl23*(×2)*, rpl32, rpl33, rpl36* |
| Other genes | Subunit of Acetyl-CoA-carboxylase | *accD* |
| Other genes | c-type cytochrom synthesis gene | *ccsA* |
| Other genes | Envelop membrane protein | *cemA* |
| Other genes | Translational initiation factor | *infA* |
| Other genes | Protease | *clpP*** |
| Other genes | Maturase | *matK* |
| Unkown | Conserved open reading frames | *ycf1, ycf2*(×2)*, ycf3****, ycf4* |

Notes: Gene *: 1-intron gene; Gene **: 2-intron gene; Gene (×2): Multi-copy(2×).

**Table S3. SSR motif distribution in *Paris polyphylla* var. *stenophylla* chloroplast genome**

| **Repeats** | **5** | **6** | **7** | **8** | **9** | **10** | **11** | **12** | **13** | **14** | **15** | **16** | **17** | **18** | **19** | **20** | **21** | **Total** | **%** | **A/T: %** |
| --- | --- | --- | --- | --- | --- | --- | --- | --- | --- | --- | --- | --- | --- | --- | --- | --- | --- | --- | --- | --- |
| A | - | - | - | - | - | 19 | 5 | 3 | 4 | 2 | - | - | - | - | - | - | - | 33 | 40.24 |  |
| C | - | - | - | - | - | - | - | 1 | - | - | - | - | - | - | - | - | - | 1 | 1.22 |  |
| T | - | - | - | - | - | 18 | 8 | 1 | 1 | 1 | 1 | - | - | - | - | - | - | 30 | 36.59 | 76.83 |
| AC | - | 1 | - | - | - | - | - | - | - | - | - | - | - | - | - | - | - | 1 | 1.22 |  |
| AT | - | 2 | 2 | - | - | - | - | - | - | - | - | - | - | - | - | - | - | 4 | 4.88 |  |
| TA | - | 3 | 2 | - | - | 1 | - | - | - | - | - | - | - | - | - | - | - | 6 | 7.32 |  |
| AAT | 1 | - | - | - | - | - | - | - | - | - | - | - | - | - | - | - | - | 1 | 1.22 |  |
| ATA | 1 | - | - | - | - | - | - | - | - | - | - | - | - | - | - | - | - | 1 | 1.22 |  |
| AATGAC | - | - | - | - | - | - | - | - | - | - | - | - | - | - | - | - | 1 | 1 | 1.22 |  |
| ATACTA | - | - | - | - | - | - | - | - | - | - | - | - | - | 1 | - | - | - | 1 | 1.22 |  |
| ATAGTA | - | - | - | - | 1 | - | - | - | - | - | - | - | - | - | - | - | - | 1 | 1.22 |  |
| GGAAGA | 1 | - | - | - | - | - | - | - | - | - | - | - | - | - | - | - | - | 1 | 1.22 |  |
| TTTATC | 1 | - | - | - | - | - | - | - | - | - | - | - | - | - | - | - | - | 1 | 1.22 |  |
| Total | 4 | 6 | 4 | 0 | 1 | 38 | 13 | 5 | 5 | 3 | 1 | 0 | 0 | 1 | 0 | 0 | 1 | 82 |  |  |
